# A preliminary examination of the diagnostic value of deep learning in hip osteoarthritis

**DOI:** 10.1371/journal.pone.0178992

**Published:** 2017-06-02

**Authors:** Yanping Xue, Rongguo Zhang, Yufeng Deng, Kuan Chen, Tao Jiang

**Affiliations:** 1 Department of Radiology, Beijing Chaoyang Hospital Affiliated to Capital Medical University, Beijing, China; 2 Infervision, Beijing, China; Chinese Academy of Sciences, CHINA

## Abstract

Hip Osteoarthritis (OA) is a common disease among the middle-aged and elderly people. Conventionally, hip OA is diagnosed by manually assessing X-ray images. This study took the hip joint as the object of observation and explored the diagnostic value of deep learning in hip osteoarthritis. A deep convolutional neural network (CNN) was trained and tested on 420 hip X-ray images to automatically diagnose hip OA. This CNN model achieved a balance of high sensitivity of 95.0% and high specificity of 90.7%, as well as an accuracy of 92.8% compared to the chief physicians. The CNN model performance is comparable to an attending physician with 10 years of experience. The results of this study indicate that deep learning has promising potential in the field of intelligent medical image diagnosis practice.

## 1. Introduction

Osteoarthritis (OA) is the most common form of arthritis that involves inflammation and major structural changes of the joint, resulting in pain and functional disability [1]. Its main symptoms, pain and stiffness, are major reasons undermining the ability of performing daily living activities, especially for the elderly. Among different types of osteoarthritis, hip osteoarthritis (hip OA) has been a substantial public health problem, causing considerable social burdens and economic costs due to work loss or related disabilities [1–6]. Therefore, it is critical to diagnose and assess hip OA in a timely and precise manner. Conventionally, hip OA is identified using X-ray imaging, which is cost-efficient, easy and rapid [7], and remains as the gold standard for the imaging-based diagnosis and evaluation of joint structural alterations [8]. X-ray images are manually assessed and scored by specific physicians, based on several radiological features, mainly the definite presence of osteophytes and the presence or absence of joint space narrowing (JSN) [8–10]. However, manual assessment is time consuming and sometimes, inaccurate. Nevertheless, with the increasing of aging population in nearly all countries, especially in China [11], the demand of diagnosis is growing ever larger than before. Thus time-efficient and accurate automatic diagnosis will become necessary in the near future.

Recently, with the progress of statistical science and information technology, researchers have been attempting to exploit deep learning as a novel method for diagnosis based on medical images. Deep Learning is an artificial intelligence method that achieves multi-layer feature learning by building deep network structures. As a branch of machine learning, deep learning has currently been broadly used on speech recognition, object detection and many other fields [12]. The conventional neural networks used in machine learning typically have one hidden layer, which are fully connected with the input layer and output layers. In comparison, deep learning is built upon deep neural networks that have multiple layers between the input layer and the output layer.

Among the architectures of deep learning, convolutional neural networks (CNN) is especially suitable for image diagnosis because it is designed to process data in the form of multiple layers, and it is able to take advantage of natural image properties, such as local connections, shared weights, pooling and the use of many layers to perform preferred analysis [13]. The key to the success of deep learning is big data sets, without which the myriad parameters in deep learning networks could not be fully trained. Many researches using CNN for medical image diagnosis have been reported, including grading of glioma [14], microscopy image classification [15], breast cancer discrimination [16], histopathological diagnosis [17], knee cartilage segmentation [18], brain registration [19], and automatic feature detection [20]. However, studies using deep learning for hip OA diagnosis have not been reported yet.

In this paper, we applied a previously validated CNN model for hip OA diagnosis and compare its outcome with conventional manual assessment. Excitingly, CNN yields a relatively decent sensitivity and even better specificity in diagnosis, which provides promising potential of automated diagnosis for this disease in the future.

## 2. Methods

**Fig 1:**
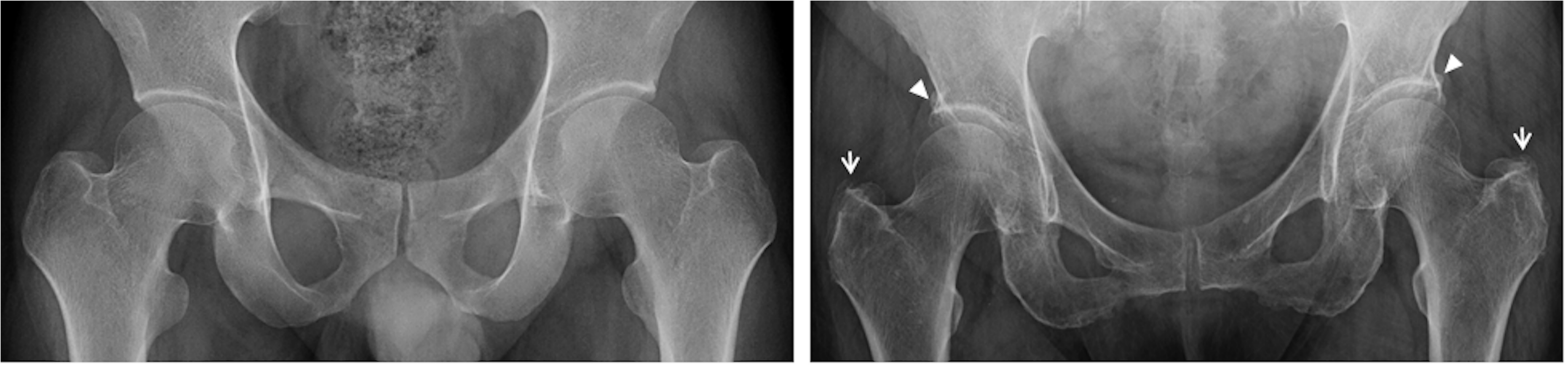
Sample normal and OA hip X-ray images. The images have a pixel resolution of 0.187 mm x 0.187 mm, and were zoomed in on the osteophytes to highlight the differences between normal and OA patients. Left: Normal hip joint X-ray image. Right: OA image. The triangles indicate osteophytes of the acetabular rim bone. The arrows indicate the osteophytes of the greater trochanters of femurs.

In this study, 420 anteroposterior (AP) view hip X-ray images acquired between March 2016 and April 2016 were collected retrospectively from Beijing Chaoyang Hospital, China. This study was approved by the local (Beijing Chaoyang Hospital) ethics committee (Chaoyang Hospital IRB: 2015–164). These X-ray images were assessed by 2 chief physicians with more than 20 years of experience, and were separated into a normal group and an OA group. The normal group had 219 samples and the OA group had 201 samples. One-fifth of the images were randomly selected as the test samples (43 from the normal group, 40 from the osteoarthritis group), and the rest were saved as the training samples. Fig 1 shows an example pair of normal and OA hip X-ray images. The raw images were 432 mm x 356 mm, with a pixel resolution of 0.187 mm x 0.187 mm. The images were zoomed in on the osteophytes to highlight the differences between normal and OA images. The arrows in the OA image indicate the positions of osteophytes and altered shape of the joint ends, which are the features typically used to diagnose hip OA in manual assessment.

**Fig 2:**
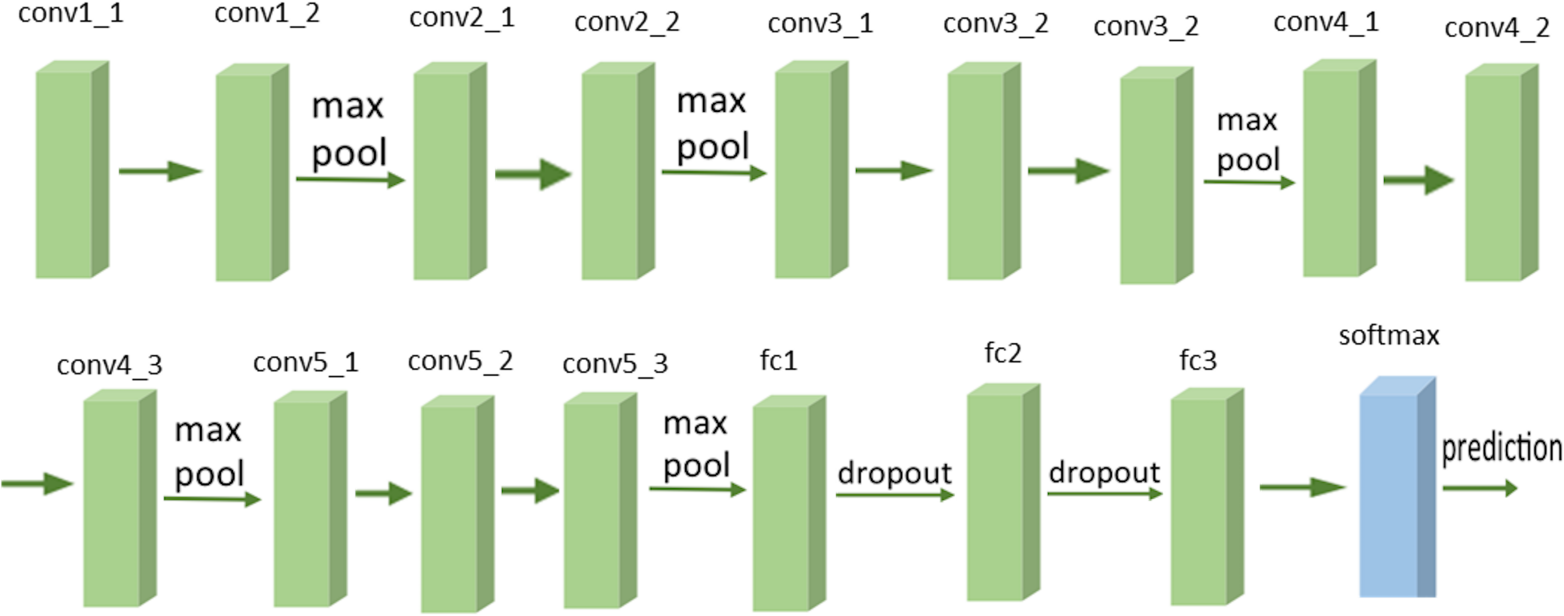
VGG-16 network block diagram. It contains 16 CONV/FC (13 convolutions and 3 fully connected) layers.

A previously validated CNN model consisting of 16 layers was trained with the training samples. This network is called the VGG-16 layer network, which was the runner-up in ILSVRC 2014 developed by Karen Simonyan and Andrew Zisserman [20]. The depth of the network was shown to be a critical component for good performance. The final best configuration contains 16 CONV/FC layers and, appealingly, features an extremely homogeneous architecture that only performs 3x3 convolutions and 2x2 pooling from the beginning to the end, which is the configuration used in this study. Fig 2 illustrates the block diagram of this network. Our implementation of the whole VGG network is composed of CONV layers that perform 3x3 convolutions with stride 1 and pad 1, and of POOL layers that perform 2x2 max pooling with stride 2 (and no padding).

**Fig 3:**
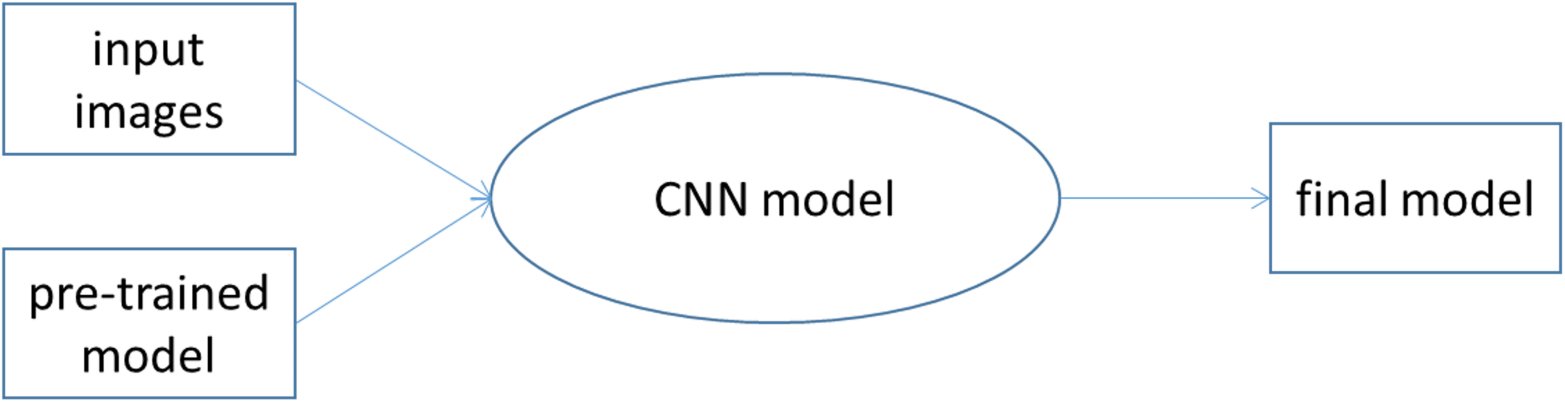
Hip joint model training flow chart.

The initial parameters in the VGG-16 layer network used this study were adopted from the ImageNet competition. We then input the 337 training X-Ray images (176 normal, 161 osteoarthritis) into the model to fine-tune the parameters using back propagation method. Fig 3 represents the training process of the deep learning network. The network was then applied to the test samples to provide an OA prediction result.

We performed cross-validation to finalize the hyper-parameters of the VGG model for this study. The data was split into 5 groups and one of the groups was used as the validation set. In the validation process, we adjust parameters (such as learning rate and dropout) to tune the final model. We achieve the final parameters based on the five-cross validation. With the parameters: learning rate = 0.0001, dropout = 0.7, the validation accuracy are 92.77%, 97.59%, 91.57%, 92.77%, 95.18%. We implemented the deep learning model using the Lasagne framework based on Theano, performed training, validation and testing on a workstation with a Nvidia Titan X GPU that has 12GB memory. Based on our implementation and our hardware configuration, the model took 0.021s to process every image during testing.

In addition, each test sample was diagnosed by a resident physician, an attending physician, and an associated chief physician (ranked from low to high, with experience level from low to high). The resident physician has 3 years of experience of hip OA diagnosis, while the attending physician has 10 years of experience and the associate chief physician has 15 years. The human diagnostic results, as well as the computer model prediction results, were compared with the diagnosis decision by the two chief physicians (more than 20 years of experience, highest ranked), whose diagnosis is considered as the baseline standard in this study. The Diagnosis Agreement Rate (DAR) was defined as the ratio of the number of matched diagnosis between 2 diagnostic decisions to the total number of the test samples.

## 3. Results

Table 1 presents the CNN model prediction results on the 83 test images. Based on these results, the deep learning model had sensitivity 38/(38+2) = 95.0% and specificity 39/(39+4) = 90.7%. The DAR between the CNN model and the chief physicians (baseline standard) is (39+38)/83 = 92.8%.

**Table 1:** The mixed table of the predicted diagnosis and the actual diagnosis.

Confusion matrix		standard	
		normal: 43	abnormal: 40
Test results	**normal: 41**	39	2
	**abnormal: 42**	4	38

The DAR between chief physicians and other groups of doctors are listed in Table 2. The DAR between attending physician and chief physician is similar to the DAR between CNN model and the chief physician.

**Table 2:** The DAR of different doctors and our deep learning method.

DAR	resident physician	attending physician	associate chief physician
Chief physician	75.9%	92.7%	96.4%
Deep learning model	74.7%	86.7%	92.8%

The DAR between the CNN model and the other human diagnoses are also listed in Table 2. The CNN model achieves higher DAR with more experienced physicians (associate chief physician and chief physician).

The DAR between different groups of physicians was also computed. The DAR between the resident physician and the attending physician was 78.3%, the DAR between the resident physician and the associate chief physician was 75.9%, and the DAR between the attending physician and the associate chief physician was 92.8%.

Table 3 is the list of the sensitivity and specificity of different doctors and the deep learning model. Compared with the three physicians, the deep learning model achieved a good balance between finding true positive cases and excluding true negative cases.

**Table 3:** The comparison of sensitivity and specificity of the deep learning model with doctors.

	resident physician	attending physician	associate physician	Deep Learning Model
Sensitivity	100%	100%	100%	95.0%
Specificity	53.5%	86.0%	93.0%	90.7%

**Fig 4:**
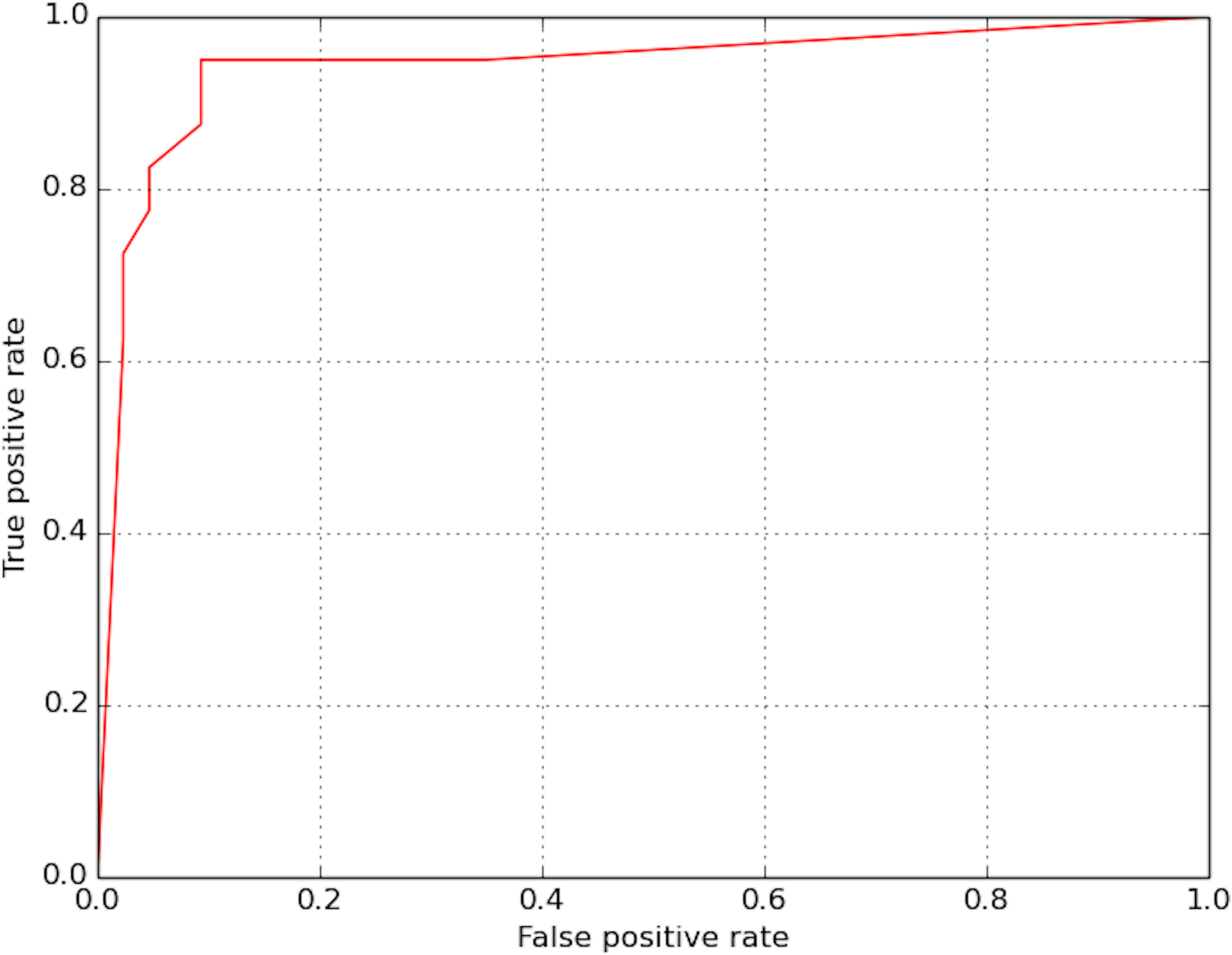
ROC curve of the CNN model.

Fig 4 shows the receiver operating characteristic (ROC) curve of our deep learning model. The area under the curve is 0.94.

**Fig 5:**
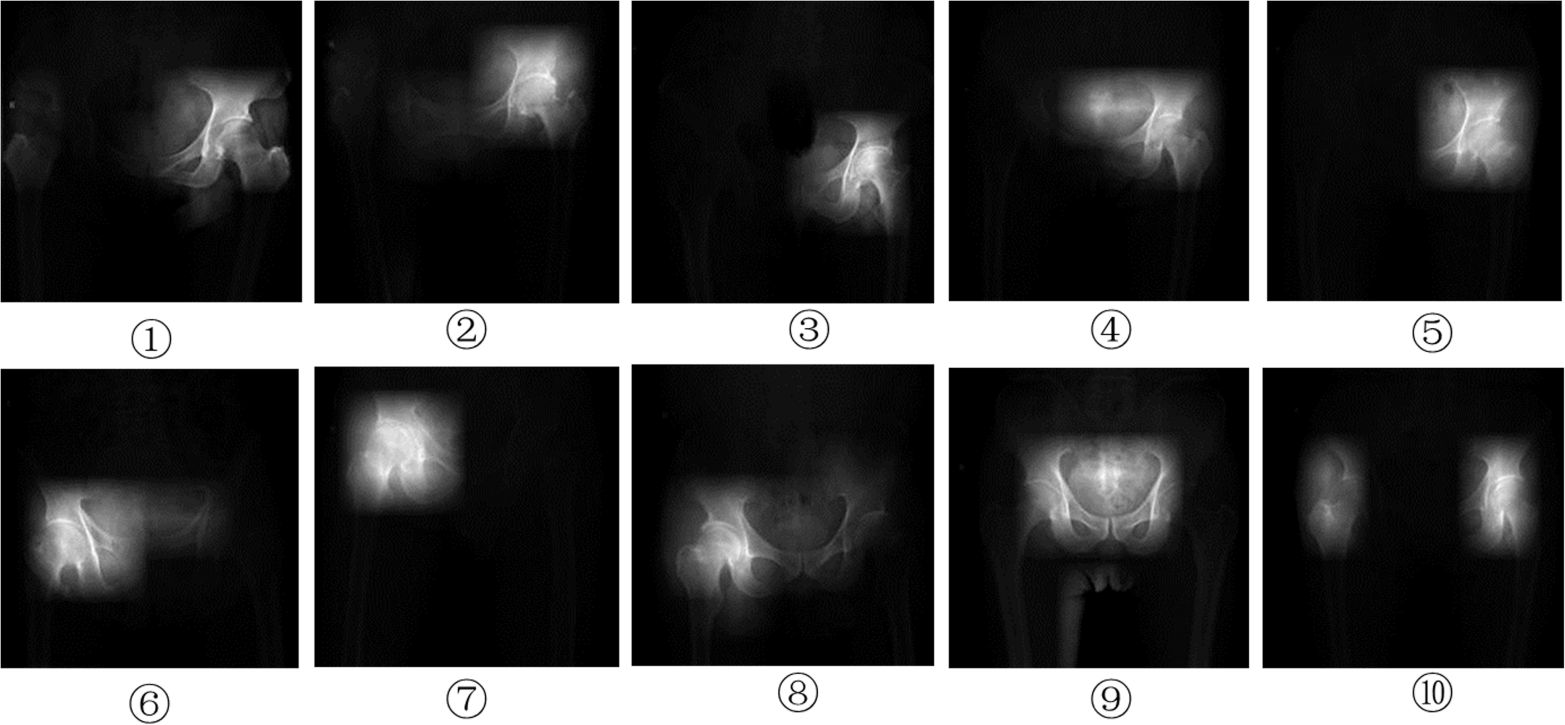
The saliency images of diseased group. They are: 1) the five images(①②③④⑤) in top row, the arthritic areas are located in the left hip joint; 2) the left three images(⑥⑦⑧) in bottom row, the arthritic areas are located in the right hip joint; 3) the right two images(⑨⑩) in bottom row, the arthritic areas are located in both hip joints.

In order to understand the exact features in the X-ray images that were assessed by the deep learning model to diagnose OA, we used the trained model to visualize the images and highlight the saliency regions. These saliency regions represent the regions of interest, where the deep learning model extracts information to make the diagnostic decision. Fig 5 shows 10 example saliency images from the diseased group. The highlighted regions in each of the 10 images correspond to the arthritic areas according to the assessment of the chief physicians. Images ①⑨show mild OA with osteophytes on the acetabular margins; Images ②③⑤⑥⑧⑩ show moderate OA with joint space narrowing (JSN), osteophytes on the femoral and acetabular margins, and subchondral sclerosis; Images ④⑦ show severe OA with gross loss of joint space with sclerosis and cysts, deformity of the femoral head and acetabulum, and large osteophytes.

## 4. Discussion

This study is a preliminary examination of the diagnostic value of deep learning in hip osteoarthritis. The deep learning algorithm used in this study, a CNN model, was able to achieve similar accuracies compared to the attending physician with 10 years of experience.

Table 1 shows the performance of our CNN model. The model achieved a diagnostic accuracy of 92.8% compared to the diagnosis provided by the most experienced chief physicians. In addition, the model achieved a good balance between high sensitivity and specificity. Comparing the accuracy of the CNN model to the accuracy of different groups of doctors listed in Table 2, the CNN model has a similar accuracy with the attending physician who had 10 years of experience.

The DAR was calculated between the deep learning model and different groups of physicians as shown in Table 2. The CNN model achieves higher DAR with more experienced physicians (92.8% compared to associate chief physician and chief physicians). This indicates the prediction outcome of the CNN model is more similar to the decision made by experienced physicians.

The DARs among different groups of physicians were also computed. The results showed that the diagnosis decisions on the same set of images could vary largely from doctors to doctors, depending on their experience levels. On the other hand, the performance of the deep learning model depends on the quality and sample size of the training data. A comprehensive and accurate data set could lead to an accurate model with great generalizability. Given the training size of 337 images (176 normal and 161 with OA) diagnosed by two experienced chief physicians, our deep learning model was able to achieve high sensitivity and specificity.

Table 3 shows the sensitivity and specificity of the CNN model and different groups of doctors. All 3 groups of doctors reached a sensitivity of 100%, while the specificity increased with increasing experience and rank of the doctors. Particularly, the resident physician had a low specificity of 74.7%. This suggests inexperienced doctors are prone to over-diagnose hip OA from X-ray images. Our hypothesis is that inexperienced doctors tend to make a positive diagnostic decision when they encounter a difficult case or when they have low confidence in their decisions. False-negative diagnosis is likely to lead to legal action being taken by those individuals affected, and potentially may decrease public reputation of the hospital. On the other hand, the CNN model achieved a higher specificity than resident and attending physicians.

Fig 5 shows the saliency regions found on the X-Ray images by the deep learning model. These regions match with the arthritic lesions that are assessed by the physicians to make the diagnostic decision, and therefore proving the diagnostic validity of the deep learning model in a visual way. It also reflects that the deep learning model extracts the relevant information from the images to our problem, and it is using the same information that human doctors use to make the diagnostic decision.

To test the robustness and stability of our deep learning model on this imaging data set, we performed random splitting of the data. As described earlier, one-fifth of the images in the dataset were randomly selected as test samples and the others were used as training samples. The random splitting was done 1000 times, i.e., the training and testing processes were conducted 1000 times, each with a different combination of the training and testing samples. The average accuracy of training is 99.64%, and the average accuracy of testing is 92.43%, indicating that our deep learning model is stable and robust.

The traditional computer assisted approach for OA diagnosis is to measure the joint space width (JSW) on an X-ray radiograph using a software interface [21] [22]. JSW can be used to assess the presence of joint space narrowing (JSN). JSN is a major indicator of OA but is not the sole deciding factor for diagnosis. This traditional computer assisted method still requires human input to fine-tune the segmentation of the joint space in order to accurately measure JSW. In addition, this method typically leads to low inter-observer agreement with low intra-class Correlation Coefficient (ICC) < 0.7, and very low OA classification agreement between difference users [22]. On the other hand, our deep learning model does not require human involvement, and is able to automatically produce a diagnostic decision based on the input radiograph. The deep learning model was shown to investigate features in the radiograph that are relevant to hip OA such as JSN and the presence of osteophytes at various locations, and was able to perform at a high level similar to a radiologist with 10 years of experience.

One major limitation of this study is that we used the diagnostic decision by two chief physicians as the baseline standard. Although the two chief physicians have many years of experience diagnosing hip OA, the validity of this study can be improved if pathological results were used as the gold standard. However, we were unable to obtain pathological information because this is a retrospective study.

Based on current test results, the diagnostic value of the deep learning method came close to the level of attending physician and associate chief physician. Overall, the CNN model has a similar accuracy compared to the attending physician. On the other hand, the diagnosis decision made by the CNN model and associate chief physician have the highest agreement rate. It indicated that deep learning had its value in the field of intelligent medical image diagnosis practice. In future studies, we will expand the size of the dataset and explore the diagnostic value of deep learning in other physiological diseases from X-rays and other imaging modalities.

## 5. Conclusions

This study applied a previously validated CNN model on 420 hip X-ray images to automatically diagnose hip OA. This CNN model achieved a high sensitivity of 95.0% and high specificity of 90.7%. The CNN model performance is comparable to an attending physician with 10 years of experience. The results of this study indicate that deep learning has promising potential in the field of intelligent medical image diagnosis practice.
